# Staging procedures fail to benefit women with borderline ovarian tumours who want to preserve fertility: a retrospective analysis of 448 cases

**DOI:** 10.1186/s12885-020-07262-w

**Published:** 2020-08-17

**Authors:** Na Li, Jinhai Gou, Lin Li, Xiu Ming, Ting Wenyi Hu, Zhengyu Li

**Affiliations:** 1 Department of Gynecology and Obstetrics, West China Second University Hospital, Sichuan University, Chengdu, Sichuan 610041 P.R. China; 2 Key Laboratory of Obstetrics & Gynecologic and Pediatric Diseases and Birth Defects of Ministry of Education, West China Second Hospital, Sichuan University, Chengdu, Sichuan 610041 P.R. China; 3 Department of Obstetrics and Gynecology, The First Affiliated Hospital of Zunyi Medical University, Zunyi, Guizhou 563000 P.R. China

**Keywords:** Borderline ovarian tumour, Surgery staging, Fertility-sparing surgery, Disease-free survival

## Abstract

**Background:**

To evaluate the effect of clinicopathologic factors on the prognosis and fertility outcomes of BOT patients.

**Methods:**

We performed a retrospective analysis of BOT patients who underwent surgical procedures in West China Second University Hospital from 2008 to 2015. The DFS outcomes, potential prognostic factors and fertility outcomes were evaluated.

**Results:**

Four hundred forty-eight patients were included; 52 recurrences were observed. Ninety-two patients undergoing FSS achieved pregnancy. No significant differences in fertility outcomes were found between the staging and unstaged surgery groups. Staging surgery was not an independent prognostic factor for DFS. Laparoscopy resulted in better prognosis than laparotomy in patients with stage I tumours and a desire for fertility preservation.

**Conclusion:**

Patients with BOT fail to benefit from surgical staging. Laparoscopy is recommended for patients with stage I disease who desire to preserve fertility. Physicians should pay more attention to risk of recurrence in patients who want to preserve fertility.

## Background

Borderline ovarian tumour (BOT) is a unique type of tumour with a better prognosis than malignant ovarian tumours. BOT usually occurs in women 10 years younger than those with epithelial ovarian cancer. The majority of the women with BOT are diagnosed in earlier stages, reported about 75% diagnosed at stage I [1, 2]. It was reported that in BOT specimens, the significant marker for malignant tumours, Ki67 Labeling Index value, ranged from 2 to 40% [3].

The clinical management of BOT has evolved since our understanding of its biological behaviour has increased over the latest two decades. The primary treatment for BOT is surgical removal of the tumour, while fertility-sparing surgery (FSS) is emphasized in women who desire to preserve their fertility. The role of comprehensive surgical staging in the treatment of BOT is still controversial. Due to that peritoneal implants are a significant prognostic index and the most common sites of implants include the omentum and peritoneal surfaces, comprehensive surgical staging including resection of the primary borderline tumour, abdominal/pelvic cytologic washings, omentectomy, and peritoneal biopsies is recommended. However, it is reported that routine lymphadenectomy is not recommended [4, 5]. In general, comprehensive surgical staging, adequate tissue sampling, and adequate follow-up period are essential aspects for optimal clinical management of BOT [2]. It is still inconsistent of the benefits of staging surgery, while a recent systematic literature review showed that staging surgery, including hysterectomy and lymphadenectomy for BOT, is not supported based on present studies [6–8]. As the ratio of uterine or nodal metastasis is low in early-stage BOT, the risk of surgical complications and the benefits of surgical staging must be balanced carefully.

To evaluate the effect of clinicopathologic factors on the prognosis and fertility outcomes of BOT patients, this study was performed.

## Methods

Clinical data of BOT patients were collected retrospectively in West China Second University Hospital between January 2008 and December 2015. Patients with a pathological diagnosis of BOT who underwent surgery were enrolled in this study. The patients with concurrent ovarian cancer, other malignant reproductive tumours, or incomplete data were excluded. This study was approved by the Medical Ethics Committee of West China Second University Hospital. Data were collected from medical records, telephone interview and out-patient review. Essential information included data of age, lesion location, International Federation of Gynecology and Obstetrics (FIGO) stage, histological subtype, surgical information, chemotherapy information, and follow-up information. Although the FIGO ovarian staging classification was revised on 1 January 2014, we used the previous staging (2009) classification guideline for consistency [9]. In addition, histological type was determined according to the World Health Organization (WHO) system (2003). Pathological specimens were evaluated by two independent pathologists experienced in gynaecologic pathology. The tumours were divided into four histological types: serous, mucinous, endometrioid, and other types. Micropapillary lesions were defined as serous tumours with complex micropapillary structures [10]. Microinvasion lesions were defined as stromal invasion limited in an area of less than 10 mm^2^ [10]. Surgical mentioned in this study included FSS, which was performed to conserve the uterus and at least one ovary, and radical resection, which was performed to remove the uterus and bilateral salpingo-oophoron [11]. Moreover, several surgery types need to be defined: staging, and non-staging surgery. Staging was defined as surgery including peritoneal washing and/or biopsies, pelvic and para-aortic lymphadenectomy (sampling or systematic), and omentectomy. Other surgery was defined as non-staging surgery [12]. Four types of FSSs are mentioned as follows: unilateral salpingo-oophorectomy, unilateral cystectomy, bilateral cystectomy, and unilateral salpingo-oophorectomy plus contralateral cystectomy. The latter three surgeries were defined as cystectomy. Patients were followed-up once every 3 months for the first 2 years, every 6 months for 3–5 years after the surgery, and once per year thereafter. Gynaecological examination, abdominal ultrasonography, and serum tumour marker evaluation, especially ca-125, were performed in each follow-up. Considering the favourable prognosis, disease-free survival (DFS, defined as the duration from the primary surgery to the first recurrence or the last follow-up) was applied to assess oncological outcomes, rather than over-all survival (OS).

DFS, recurrence rate, and pregnancy rate were selected as the primary outcomes in this study. All statistical analyses were performed using the Statistical Package for Social Sciences (SPSS) statistical software (version 20.0). The Student’s *t*-test was used for statistical analysis of unpaired data. Univariate and multivariate Cox regression analysis were used to determine the factors affecting recurrence. A *P*-Value < 0.05 was considered statistically significant.

## Results

### Patient characteristics

A total of 448 patients with BOT were enrolled in this study. The demographics and clinicopathological characteristics are shown in Table 1.

**Table 1:** Demographics of patients with borderline ovarian tumors

	Non-staging surgery	Staging surgery	***P*** Value
Total	330	118	
Age (y, mean ± Std)	36.75 ± 14.35	38.03 ± 12.49	0.363
Time of operation(h, mean)	127.50	255.00	< 0.001
Blood Loss (ml, median)	80	400	< 0.001
Length of stay (d, median)	6	8	< 0.001
FIGO Stage			
I	287 (87%)	60 (50.8%)	< 0.001
II	8 (2.40%)	12 (10.2%)	
III	32 (9.7%)	42 (35.6%)	
IV	3 (0.9%)	4 (3.4%)	
Histology			0.038
Serous	177 (53.6%)	81 (68.6%)	
Mucinous	119 (36.1%)	31 (26.3%)	
Endometrioid	7 (2.1%)	1 (0.8%)	
Serous and Mucinous	27 (8.2%)	5 (4.2%)	
Lesion lateral			< 0.001
Unilateral	277 (83.9%)	75 (63.6%)	
Bilateral	53 (16.1%)	43 (36.4%)	
Micropapillary			
Yes	45 (13.6%)	36 (30.5%)	< 0.001
No	285 (86.4%)	82 (69.5%)	
Microinvasion			< 0.001
Yes	31 (9.4%)	57 (48.3%)	
No	299 (90.6%)	61 (51.7%)	
Carcinogenesis			
Yes	14 (4.2%)	11 (9.3%)	0.058
No	316 (95.8%)	107 (90.7%)	
Surgical Approach			< 0.001
Laparotomy	192 (58.2%)	106 (89.8%)	
Laparoscopy	138 (41.8%)	12 (10.2%)	
Ascites/Cytologic washings			0.012
Positive	14 (4.2%)	13 (11.0%)	
Negative	316 (95.8%)	105 (89.0%)	
Lymph node involvement			NA
Yes	NA	21 (18.6%)	
No	NA	92 (81.4%)	
Appendix metastasis			
Yes	6 (54.5%)	5 (45.5%)	0.05
No	113 (81.3%)	26 (18.7%)	
Omentum metastasis			NA
Yes	NA	27 (23.1%)	
No	NA	90 (76.9%)	
Adjuvant chemotherapy			< 0.001
Yes	54 (16.4%)	67 (56.8%)	
No	276 (83.6%)	51 (43.2%)	
Recurrence			0.007
Yes	30 (9.1%)	22 (18.6%)	
No	300 (90.9%)	96 (81.4%)	
Fertility-sparing surgery			< 0.001
Yes	240 (72.7%)	30 (25.4%)	
No	90 (27.3%)	88 (74.6%)	
Achieving pregnancy			0.552
Yes	79 (35.7%)	13 (41.9%)	
No	142 (64.3%)	18 (58.1%)	

Data were recorded as number (%), mean (±SD), or median (range)

*Abbreviations*: *y* Years, *h* Hours, *d* Days

The median age at diagnosis was 37.1 years (range: 11–82 years). The majority of the patients were in FIGO stage I (*n* = 347, 77.46%), with a few cases of stage II (*n* = 20, 4.46%), stage III (*n* = 74, 16.52%), and stage IV (*n* = 7, 1.56%). The most common pathological type of BOT was serous (*n* = 258, 57.59%), followed by mucinous (*n* = 150, 33.48%), serous/mucinous (*n* = 32, 7.14%), and endometrioid (*n* = 8, 1.79%). Notably, most patients had unilateral lesions (*n* = 352, 78.57%), whereas 96 (21.43%) patients had bilateral lesions. Among the patients enrolled, 81 (18.08%) had micropapillary lesions, 88 (19.64%) had microinvasion lesions, and 25 (5.58%) had carcinogenesis lesions.

Regarding surgical approach, 298 patients (66.52%) underwent laparotomy and 150 patients (33.48%) underwent laparoscopy; 118 patients (26.34%) underwent staging surgery, whereas the rest underwent non-staging surgery (330 patients, 73.66%). Abdominal/pelvic washings or ascites were collected prior to surgery for all patients, and positive involvement was identified in 27 patients (6.03%). Lymph node metastasis was detected in 21 of 113 patients (18.58%) who underwent lymphadenectomy. Appendix metastases were detected in 11 of 150 patients (7.33%) who underwent appendectomy. Omentum metastases were detected in 27 of 117 patients (23.08%) who underwent omentectomy. A total of 121 patients (27.01%) received adjuvant chemotherapy for lymph node metastasis, positive abdominal/pelvic washings, invasive implants, and/or other high-risk indicators.

### Oncological outcomes of BOT patients

We carried out a survival analysis. The median follow-up for this study was 113 (range: 14–166) months. At the last follow-up, 42 (11.6%) patients experienced recurrence, with a mean recurrence interval of 80.2 months, and 4 (0.9%) disease-specific deaths were observed. The recurrence rate in patients who underwent non-staging surgery (30/330, 9.09%) was lower than that in those underwent staging surgery (22/118, 18.64%), with the difference being statistically significant (*P* < 0.01). The results of univariate and multivariate analyses of DFS in all patients are shown in Table 2.

**Table 2:** Univariate and multivariate analysis of DFS

	Univariate		***P*** value	Multivariate		***P*** value
	HR	95% confidence interval		HR	95% confidence interval	
FIGO Stage						
I	1					
≧II	7.204	4.093–12.680	0.000	6.544	2.137–20.041	0.001
Histology						
Serous	1					0.528
Mucinous	0.353	0.171–0.726	0.005	1.215	0.275–5.375	0.797
Others	0.286	0.069–1.183	0.084	0.632	0.130–3.066	0.569
Lesion lateral						
Unilateral	1					
Bilateral	2.554	1.460–4.469	0.001	1.076	0.526–2.202	0.840
Micropapillary						
Yes	1.557	0.831–2.917	0.167			
No	1					
Microinvasion						
Yes	5.092	2.954–8.779	0.000	0.478	0.181–1.261	0.136
No	1					
Carcinogenesis						
Yes	1.049	0.327–3.366	0.936			NA
No	1					
Staging surgery						
Yes	2.191	1.263–3.801	0.005	0.810	0.393–1.669	0.567
No	1					
Adjuvant chemotherapy						
Yes	5.281	3.002–9.289	0.000	2.031	0.913–4.519	0.083
No	1					
Ascites/Pelvic washings						
Positive	5.442	2.850–10.391	0.000	3.259	1.202–8.835	0.020
Negative	1					
Surgical Approach						
Laparotomy	1					
Laparoscopy	0.292	0.132–0.647	0.002	0.319	0.128–0.793	0.014
CA-125						
Normal	1					
Elevated	2.201	1.224–3.960	0.008	0.825	0.422–1.611	0.572
Fertility sparing surgery						
No	1					
Yes	1.055	0.063–1.845	0.851			NA
Appendectomy						
No	1					
Yes	0.394	0.192–0.808	0.011			NA
Invasive implants						
No	1					
Yes	4.105	2.222–7.583	0.000	0.566	0.208–1.539	0.265

According to the univariate analysis, patients who underwent staging surgery had shorter DFS than those who underwent non-staging surgery. In addition, laparoscopy was strongly associated with improved DFS (HR = 0.292, 95% CI: 0.132–0.647, *P* = 0.002) compared to laparotomy. Other factors found to be associated with DFS were FIGO stage, histology, lesion location, microinvasion, adjuvant chemotherapy, ascites/pelvic washings, cancer antigen (CA)-125 level, appendectomy, and invasive implants (all *P* < 0.01). Micropapillary and carcinogenic lesions were not associated with DFS (*P* > 0.05).

Although several factors were found to be associated with DFS by univariate analysis, only FIGO stage (OR: 6.544, 95% CI: 2.137–20.041), positive ascites/pelvic washings (OR: 3.259, 95% CI: 1.202–8.835), and surgical approach (OR: 0.319, 95% CI: 0.128–0.793) were significantly associated with DFS (*P* < 0.001, *P* = 0.014, *P* = 0.043, respectively) as per multivariate analysis; complete staging surgery was not associated with DFS (*P* = 0.600) as per multivariate analysis. There was no difference in DFS between patients who underwent FSS and radical surgery according to univariate and multivariate analyses.

Subgroup analysis showed that in patients who underwent staging surgery, there was no difference in DFS between those who underwent laparotomy or laparoscopy (*P* = 0.349). Among patients who underwent non-staging surgery, the DFS was longer for patients who underwent laparoscopy than for those who underwent laparotomy (*P* = 0.011; Supplementary Table 1).

### Oncological outcomes in patients with BOT after FSS

Among the patients enrolled, 270 patients underwent FSS. Of these, 32 patients (11.8%) experienced recurrence. To explore the potential risk factors associated with improved DFS in patients who underwent FSS, univariate and multivariate analyses were performed (Table 3).

**Table 3:** Univariate and multivariate analysis of DFS in fertility desiring patients after fertility-sparing surgery

	Univariate		***P*** Value	Multivariate		***P*** Value
	OR	95% confidence interval		OR	95% confidence interval	
FIGO Stage						
I	1					
≧II	21.061	9.662–45.909	0.000	11.586	4.535–29.602	**0.000**
Histology						
Serous	1		0.010			0.155
Mucinous	0.196	0.068–0.654	0.003			0.189
Others	0.000		0.975			NA
Lesion lateral						
Unilateral	1					
Bilateral	5.491	2.570–11.73	0.000	2.581	1.061–6.283	0.037
Micropapillary						
Yes	1.976	0.840–4.649	0.119			NA
No	1					
Microinvasion						
Yes	14.644	6.940–30.903	0.000			0.955
No	1					
Carcinogenesis						
Yes	0.609	0.083–4.483	0.626			NA
No	1					
Staging surgery						
Yes	4.290	1.979–9.298	0.000			0.358
No	1					
Adjuvant chemotherapy						
Yes	7.797	3.648–16.664	0.000			0.391
No	1					
Ascites/Pelivic washings						
Positive	13.350	5.612–31.770	0.000			0.888
Negative	1					
Surgical Approach						
Laparotomy	1					
Laparoscopy	0.332	0.135–0.820	0.017	0.367	0.148–0.913	0.031
CA-125						
Normal	1					
Elevated	1.649	0.748–3.632	0.215			NA
Fertility sparing surgery						
Cystectomy-included	1					
Adnexectomy	0.382	0.168–0.867	0.021	0.367	0.148–0.913	0.014
Appendectomy						
No	1					
Yes	0.240	0.083–0.692	0.008			0.189
Invasive implants						
No	1					
Yes	14.289	6.400–31.902	0.000	4.832	1.663–14.037	0.004

Univariate analysis with patients who underwent FSS showed that patients who underwent staging surgery had shorter DFS than those who underwent non-staging procedures (OR: 4.290, 95% CI: 1.979–9.298, *P* < 0.001). DFS was better among patients who underwent laparoscopy (OR: 0.332, 95% CI: 0.135–0.820, *P* = 0.017) than among those who underwent laparotomy. In addition, patients who underwent salpingo-oophorectomy had longer DFS than those who underwent a cystectomy procedure (OR: 0.230, 95% CI: 0.168–0.867, *P* = 0.021). Other factors were also associated with DFS in patients who underwent FSS, including FIGO stage, histology, lesion location, microinvasion, adjuvant chemotherapy, positive ascites/pelvic washings, appendectomy, and invasive implants *(P <* 0.05).

In multivariate analysis, there was no difference in DFS between patients who underwent staging and non-staging surgery *(P =* 0.358). There was no difference in DFS between patients with different histological types. Early FIGO stage (OR: 11.586, 95% CI: 4.535–29.602), unilateral lesions (OR: 2.581, 95% CI: 1.061–6.283), laparoscopy (OR: 0.367, 95% CI: 0.148–0.913), salpingo-oophorectomy (OR: 0.367, 95% CI: 0.148–0.913), and no invasive implants (OR: 4.832, 95% CI: 1.663–14.037) were independent factors for improved DFS (*P* < 0.05).

### Reproductive outcomes in patients with BOT after FFS

At the last follow-up, of the 270 patients who underwent FSS, 252 patients had attempted to conceive and 92 achieved pregnancy. The correlation between clinicopathological characteristics and reproductive outcome is shown in Table 4. The pregnancy rate in patients aged < 35 years was higher than those aged ≧35, at a statistically significant *(P <* 0.001) level. Of the 30 patients who underwent staging surgery, 13 patients (43.33%) succeeded in conceiving, whereas 79 of 203 patients (38.92%) who underwent non-staging surgery succeeded in conceiving, but these differences were not statistically significant *(P >* 0.05). There was no difference between patients who underwent laparotomy or laparoscopy. Similarly, among patients who underwent salpingo-oophorectomy or cystectomy, there was no difference in the pregnancy rates (*P >* 0.05).

**Table 4:** Correlation between pregnant outcomes and clinicopathological indexes in patients after fertility-sparing surgery

	Fertility outcome		***P*** value
	No (n,%)	Yes (n,%)	
Staging surgery			
No	124 (87.9)	79 (85.9)	0.691
Yes	17 (12.1)	13 (14.1)	
Surgical approach			
Laparoscopy	65 (46.1)	37 (40.2)	0.419
Laparotomy	76 (53.9)	55 (59.8)	
Surgical procedure			
Cystectomy	76 (53.9)	41 (44.6)	0.181
Salpingo-oophorectomy	65 (46.1)	51 (55.4)	
Adjuvant chemotherapy			
No	110 (78.0)	77 (83.7)	0.316
Yes	31 (22.0)	15 (16.3)	
FIGO Stage			
I	121 (85.8)	84 (91.3)	0.225
≧II	20 (14.2)	8 (8.7)	
Histology			
Serous	79 (56.0)	39 (42.4)	0.08
Mucinous	47 (33.3)	44 (47.8)	
Others	15 (10.6)	9 (9.8)	
Lesion lateral			
Unilateral	122 (86.5)	82 (89.1)	0.686
Bilateral	19 (13.5)	10 (10.9)	
Micropapillary			
No	23 (16.3)	12 (13.0)	0.576
Yes	118 (83.7)	80 (87.0)	
Microinvasion			
No	126 (89.4)	85 (92.4)	0.499
Yes	15 (10.6)	7 (7.6)	
Carcinogenesis			
No	134 (95.0)	86 (93.5)	0.771
Yes	7 (5.0)	6 (6.5)	
Ascites/Pelvic washings			
Positive	7 (5.0)	3 (3.3)	0.744
Negative	134 (95.0)	89 (96.7)	
CA-125			
Normal	82 (65.6)	60 (69.8)	0.553
Elevated	43 (34.4)	26 (30.2)	
Invasive implants			
No	132 (93.6)	88 (95.7)	0.574
Yes	9 (6.4)	4 (4.3)	
Age			
< 35	107 (75.9)	92 (100)	0.000
≧35	34 (24.1)	0	

## Discussion

In the present study, we performed a retrospective analysis of 448 patients with BOT in a single centre in China. BOTs are ovarian neoplasms with characteristics of benign or malignant tumours, frequently occurring in young women and associated with favourable prognosis. Within the past two decades, we have begun to understand the biological behaviour of BOTs; however, the optimal therapy for this disease is still controversial. Numerous studies have focused on the oncological and reproductive outcomes of BOT. In the literature, the primary points of discussion regarding BOT include the prognostic factors for overall survival (OS) or DFS, necessity of staging surgery, application of minimally invasive approaches, and outcome of conservative surgery.

Complete staging surgery generally includes resection of the primary borderline tumour (cystectomy or salpingo-oophorectomy), cytologic washings, omentectomy, peritoneal biopsies, and routine lymphadenectomy. Unlike in ovarian cancer, previous studies have shown that the prognosis of patients with BOT is generally favourable, with very low mortality [13, 14]. A Turkish Gynaecologic Oncology Group (GOG) study showed that the five-year survival rate of patients with BOT was 100%, and the median survival time was 120 months [15]. Therefore, DFS and recurrence-free survival (RFS) were defined as the main oncological outcomes. In the present study, complete staging surgery was performed in 26.3% of the patients. Although univariate analysis showed that patients who underwent staging surgery had shorter DFS than those who underwent non-staging surgery, no significant difference was found in the DFS between different surgical approaches as per multivariate analysis. These results were similar to those of previous studies [2, 12, 15–17]. The Turkish GOG study showed that comprehensive surgical staging did not lead to any difference in survival [15]. A retrospective multicentre study showed that there were no differences in the five-year RFS and OS between patients who did and did not undergo complete surgical staging [18]. Another multicentre study showed that surgical staging were not beneficial in the management of BOT [12]. A third multicentre study from Turkey that focused on mucinous BOT showed that radical surgery, omentectomy, appendectomy, and lymphadenectomy were not independent prognostic factors for progression-free survival and OS [17].

Regarding the correlation between lymphadenectomy and DFS, lymph node involvement does not appear to be a prognostic factor [19, 20]. Univariate analysis by Matsuo et al. showed that surgical staging patterns for hysterectomy and lymphadenectomy were not associated with cause-specific survival (*P =* 0.19) [2]. A previous study by Qian et al. showed that there were no significant differences between groups with or without lymphatic node involvement (*P* = 0.778), and between patients who had more or fewer than 10 nodes removed (*P =* 0.549) [16].

BOT occurs in women of all ages, with a high proportion in the reproductive age [21]. In the present study, the median age at diagnosis was 37.1 years. Therefore, a conservative surgical approach (FSS) was the preferred choice for patients who desired to preserve their fertility. However, the balance between oncological and reproductive outcomes should be assessed adequately; approximately 12–36% of the patients with BOT who undergo FSS experience recurrence [21], and the most common site of recurrence is the residual ovary [21–24]. Previous studies have shown that the recurrence rate of BOT in patients who underwent FSS was markedly higher than that in patients who underwent radical surgeries (21.4% vs. 6.3%, *P* < 0.05) [10, 25]. Furthermore, a large proportion of patients who underwent FSS experienced invasive recurrence [14]. In a recent retrospective study, patients with FSS developed more relapse than patients with radical surgeries [26]. In the multivariate analyses, fertility preservation and micropapillary pattern were independently associated with adverse disease-free survival (*P* = 0.001, 0.03 and 0.026, respectively) [26]. Regarding surgical patterns, a meta-analysis showed that unilateral cystectomy is significantly associated with high recurrence rates [11]. However, another study reported that there was no statistically significant difference between patients who underwent cystectomy or unilateral salpingo-oophorectomy [27]. A recent study involving 6295 patients showed that FSS was associated with worse DFS in patients aged ≥50 years than in those aged < 50 years [28]. Another study showed that surgical procedure (conservative vs. radical) was not an independent prognostic factor for DFS or OS [12].

In the present study, both univariate and multivariate analyses results showed no significant difference in the DFS between patients who underwent FSS and those who did not (*P >* 0.05). In patients who underwent FSS, there was no significant difference in DFS between those who underwent staging and those who did not (*P >* 0.05), whereas a significant difference was observed between those who underwent laparoscopy and laparotomy (*P <* 0.05). However, no significant differences were found in the reproductive rates of those who underwent staging surgery or a different surgical approach. Therefore, the balance between oncological and reproductive outcomes in patients of reproductive age should be considered before performing FSS.

The standard treatment for BOT is surgery. Since most patients are of childbearing age, surgeons should consider using a minimally invasive procedure. Laparoscopic surgery has several advantages over open surgery in the management of gynaecologic diseases, including fewer peri-operative complications and superior cosmetic outcomes. In this study, approximately 33.48% of the patients underwent laparoscopic surgery. As per both univariate and multivariate analyses findings, laparoscopic surgery was more positively associated with improved DFS than laparotomy (*P <* 0.05). Similarly, a previous study by Song et al. also showed that RFS and OS did not differ between the laparoscopy (single-port and multi-port laparoscopy) and laparotomy groups [29]. However, the potential selective bias should be noticed, which means that the characteristic of individual patients might influence the surgery approach. For those patients with smaller mass, younger ages, lower CA125 levels in pre-operative time, laparoscopy may be more favorable, usually getting a better prognosis. However, for those patients with larger mass, older ages, higher CA125 levels, or other signs suspected for malignant tumors in pre-operative time, laparotomy was possibly chosen. This bias could be solved through increasing patients enrolled, or randomized controlled trial.

In a retrospective study of 1069 patients with BOT in Japan, 49% had normal serum CA-125 levels and only 23% had serum CA-125 levels above 100 U/mL [21]. In another study of 198 patients in Singapore, the preoperative serum CA-125 levels of 77 (39%) patients were > 35 U/mL [30]. In the present study, the serum level of CA-125 was not an independent prognostic factor for patients with BOT after FSS.

Because an accurate intra-operative diagnosis is important in the management of BOT, frozen-section examination should be performed to help surgeons and patients’ families make decisions during intra-operative periods. The accuracy of frozen-section examination is lower than optimal and the availability of reliable frozen-section analysis methods in many hospitals is difficult. Previous studies have shown that the matched rate between the results of frozen-section and definitive histological examination varies from 66.67 to 88.9% [31, 32]. Therefore, it is important for surgeons to counsel patients and their families with regard to possible intra-operative indications.

## Conclusions

Patients with BOT do not benefit from surgical staging procedures in terms of prognosis and fertility outcomes. Laparoscopy, rather than laparotomy, should be recommended for patients with stage I disease who wish to preserve their fertility. In addition, patients with advanced stage disease, invasive implants, and/or bilateral tumours who wish to maintain their fertility should consider the risk of recurrence before choosing FSS. Unilateral salpingo-oophorectomy is an alternative method for patients with BOT to preserve their fertility.

## Supplementary information

**Additional file 1: Table S1.** Subgroup analysis of staging surgery in DFS of patients undergoing laparoscopy or laparotomy.

## Data Availability

The datasets used and analysed during the current study available from the corresponding author on reasonable request.

## Abbreviations

BOT: Borderline ovarian tumour
DFS: Disease-free survival
FIGO: Federation of Gynecology and Obstetrics
FSS: Fertility-sparing surgery
GOG: Gynaecologic Oncology Group
HR: Hazard ratios
OS: Overall survival
RFS: Recurrence-free survival
SPSS: Statistical Package for Social Sciences
WHO: World Health Organization
