# Supplementary material for: Staging procedures fail to benefit women with borderline ovarian tumours who want to preserve fertility: a retrospective analysis of 448 cases
Source: BMC Cancer. 2020 Aug 17;20:769. doi: 10.1186/s12885-020-07262-w (PMC7433083; doi:10.1186/s12885-020-07262-w)
Supplement: Supplementary file 1 — Additional file 1: Table S1. Subgroup analysis of staging surgery in DFS of patients undergoing laparoscopy or laparotomy. [file 12885_2020_7262_MOESM1_ESM.doc]

|  | **Laparoscopy** | |  | **Laparotomy** | |  | **P Valve** |
| --- | --- | --- | --- | --- | --- | --- | --- |
|  | **mean** | **95%**  **Confidence interval** |  | **mean** | **95%**  **Confidence interval** |  |
| **Complete staging** | 120.167±8.457 | 103.590-136.743 |  | 107.379±4.079 | 99.385-115.373 |  | 0.349 |
| **Incomplete staging/unstaged** | 150.920±2.430 | 146.158-155.683 |  | 141.914±3.478 | 135.098-148.731 |  | 0.011 |

**Supplementary table1**

**Subgroup analysis of staging surgery in DFS of patients undergoing laparoscopy or laparotomy**
